# ID 101 - Derivados da *Cannabis* e seus Análogos Sintéticos para o Tratamento da Epilepsia Refratária: revisão sistemática de ensaios clínicos randomizados

**DOI:** 10.5327/2237-9622.2025.v34s1.101

**Published:** 2025-11-25

**Authors:** Ana Luiza Cabrera Martimbianco, Roberta Borges Silva, Carolina de Oliveira Cruz Latorraca, Isabela Porto de Toledo, Isabela Porto de Toledo, Verônica Colpani, Rachel Riera

**Keywords:** epilepsia refratária, *Cannabis*, canabidiol, revisão sistemática

## Abstract

**Introdução:**

Segundo a Organização Mundial da Saúde (OMS), cerca de 5 milhões de pessoas são diagnosticadas com epilepsia em todo o mundo. A persistência de crises epiléticas mediante tratamento com pelo menos dois medicamentos é recorrente, e leva a crises convulsivas frequentes e graves. Os derivados da e seus análogos sintéticos têm sido amplamente investigados como alternativas terapêuticas devido ao potencial benefício na redução de crises convulsivas. O objetivo deste estudo foi identificar, avaliar e sumarizar as evidências científicas disponíveis acerca dos benefícios e dos riscos dos derivados da e seus análogos sintéticos para o tratamento de epilepsia refratária.

**Método:**

Revisão sistemática conduzida no Nats/NeV do Hospital Sírio-Libanês, seguindo as recomendações metodológicas do e relatada de acordo com o (PRISMA 2020). O protocolo foi registrado prospectivamente na base PROSPERO (https://www.crd.york.ac.uk/prospero/display_record.php?ID=CRD42024499388). Foi realizada busca ampla e sensível em fevereiro de 2024, em bases eletrônicas (CENTRAL, Embase, LILACS, MEDLINE), literatura cinzenta (DANS), registros de ensaios clínicos (clinicaltrials.gov e ICTRP-WHO) e busca manual em listas de referências. Foram considerados ensaios clínicos randomizados (ECR) que avaliaram os derivados da e seus análogos sintéticos, comparado com placebo, tratamento padrão, ou qualquer tratamento ativo. Desfechos primários: frequência e gravidade de crises convulsivas, eventos adversos graves; secundários: qualquer evento adverso, qualidade de vida, função cognitiva, saúde mental. O risco de viés foi avaliado com a tabela de risco de viés da Cochrane e a certeza da evidência, pela abordagem GRADE.

**Resultados:**

Sete ECR foram incluídos, a maioria com baixo risco de viés. Foi observado que o canabidiol (CBD) 20 mg/kg/dia e 10 mg/kg/dia provavelmente aumenta a frequência de pacientes que atingiram pelo menos 50% de redução mensal de crises convulsivas (20 mg/kg/dia: Risco Relativo [RR] 1,92; Intervalo de Confiança de 95% [IC 95%] 1,49 a 2,46, n=575, 4 ECR; 10 mg/kg/dia: RR 1,94; IC 95% 1,32 a 2,86, n=280, 2 ECR, certeza moderada da evidência). A incidência de eventos adversos graves é provavelmente maior com CBD 20 mg/kg/dia (RR 2,30; IC 95% 1,36 a 3,89, n=583, 4 ECR, certeza moderada da evidência), e pode ser maior com CBD 10mg/kg/dia (RR 1,62; IC 95% 0,92 a 2,84, n=272, 2 ECR; baixa certeza da evidência). O CBD 20 mg/kg/dia provavelmente resulta em maior frequência de qualquer evento adverso (RR 1,19; IC 95% 1,04 a 1,36; n=583, 4 ECR, certeza moderada da evidência); CBD 10 mg/kg/dia pode resultar em pouca ou nenhuma diferença (RR= 1,05, IC 95% 0,89 a 1,24, n=272, 2 ECR, baixa certeza da evidência). Há incerteza quanto aos efeitos da intervenção com relação à gravidade das crises, à qualidade de vida, à função cognitiva e à saúde mental (evidência de certeza muito baixa).

**Conclusão:**

Os achados desta revisão apontam para o provável efeito do CBD 20 mg/kg/dia e 10 mg/kg/dia na redução mensal mínima de 50% nas crises convulsivas para pessoas com epilepsia refratária. A incidência de eventos adversos graves parece aumentar com a maioria dos esquemas terapêuticos analisados. Futuros ECR com maior tamanho amostral podem contribuir para a melhor tomada de decisão quanto ao uso dos derivados da e seus análogos sintéticos para epilepsia refratária.

